# Lee *et al*., Inhibitory Effect and Mechanism of Antiproliferation of Isoatriplicolide Tiglate (PCAC) from *Paulownia Coreana*. *Molecules* 2012, *17*, 5945-5951: A Note Regarding *Paulownia coreana.*

**DOI:** 10.3390/molecules18032587

**Published:** 2013-02-26

**Authors:** Christophe Wiart

**Affiliations:** School of Biomedical Sciences, University of Nottingham, Medical School, Queen’s Medical Centre, Nottingham NG7 2UH, UK; E-Mail: Christophe.Wiart@nottingham.edu.my

I read with interest the article by Lee *et al.* entitled “Inhibitory Effect and Mechanism of Antiproliferation of Isoatriplicolide Tiglate (PCAC) from *Paulownia Coreana*” [1]. This article is quite interesting and the authors should be complimented for the significant amount of work they have done. The purpose of this letter is to call attention to the need for some clarification on the name of the plant described in this article. Lee *et al.* state: “*Paulownia coreana* has traditionally been used as the medicine and health food in the treatment of cancer and infectious diseases.” and elsewhere: “In fact, many cancer research studies have been conducted using traditional medicinal plants such as *P. coreana*.” [1]. 

I have been studying the pharmacotoxicological properties of the medicinal plants of Asia and the Pacific for the last 15 years [2,3,4,5,6,7] and the sole members of the genus *Paulownia* Siebold & Zucc. (1835) officially recognized in China and Korea are *Paulownia catalpifolia* T. Gong ex D.Y. Hong, *Paulownia elongata* S.Y. Hu, *Paulownia fargesii* Franch., *Paulownia fortunei* (Seem.) Hemsl., *Paulownia kawakamii* T. Itô, *Paulownia taiwaniana* T.W. Hu & H.J. Chang and *Paulownia tomentosa* (Thunb.) Steud. [8]. *Paulownia coreana* does not exist and therefore cannot be medicinal nor the source of the phytochemical described in [1]. In addition, the chemical structure of isoatriplicolide tiglate as provided by Lee *et al*. is incomplete as regards to carbon numbering and the class of sesquiterpene to which this molecule belongs is not specified. Prompt revision of the name assigned to this plant (and henceforth any comments on its properties or phytochemical content) is required to ensure the accuracy of the scientific record.
